# Effect of a poloxamer-based thermosensitive gel on rotator cuff repair in a rabbit model: a controlled laboratory study

**DOI:** 10.1186/s13018-019-1246-2

**Published:** 2019-06-25

**Authors:** Tae In Kim, Whanik Jung, Jin-Young Chung, Hyunseok Jeong, Sae Hoon Kim

**Affiliations:** 1 Department of Orthopedic Surgery, Seoul National University College of Medicine, Seoul National University Hospital, Seoul, South Korea; 2 Department of Veterinary Internal Medicine and Institute of Veterinary Science, College of Veterinary Medicine, Kangwon National University, Chuncheon-si, Gangwon-do South Korea

**Keywords:** Postoperative fibrosis, Anti-adhesive agent, Poloxamer-based thermosensitive gel, Postoperative stiffness, Rotator cuff repair, Healing of the rotator cuff tendon

## Abstract

**Background:**

A common complication after rotator cuff repair is postoperative stiffness, which can be reduced by a simple application of an anti-adhesive agent. However, anti-adhesive agents may affect rotator cuff healing by preventing fibrosis. This experimental animal study evaluated the effect of the application of a poloxamer-based thermosensitive anti-adhesive gel and its influence on the healing of an acute rotator cuff repair in a rabbit model.

**Methods:**

Acute rotator cuff repair (supraspinatus tendon) was performed using a transosseous suturing method. One shoulder on a randomly selected side was treated with a local application of the anti-adhesive agent (applied side), and saline was applied to the contralateral side (control side). Biomechanical testing and histological analyses were performed at 4 and 8 weeks postoperatively. Eight rabbits were included for each testing and time point, for a total of 32 rabbits.

**Results:**

The failure load at 4 weeks was lower in the experimental group (95.2 ± 19.6 N vs. 110.0 ± 20.5 N; *P* = 0.017). Conversely, at 8 weeks, the failure load was higher in the experimental group (148.3 ± 16.2 N) than in the control group (122.4 ± 16.9 N; *P* = 0.002). Histological analyses revealed no statistically significant differences in the tendon maturing scores at 4 and 8 weeks between the two groups (all *P* > 0.05). The thickness of the fibrosis between the rotator cuff tendon and deltoid was thinner in the experimental group at both time points (0.50 ± 0.25 vs. 1.27 ± 0.47; *P* = 0.002 at 4 weeks, and 0.37 ± 0.35 vs. 1.39 ± 0.50; *P* = 0.003 at 8 weeks).

**Conclusions:**

Application of an anti-adhesive agent in this rotator cuff model confirmed the agent’s effectiveness at reducing fibrosis in the subacromial space. The healing of the tendon showed interesting results, as the experimental group had poorer biomechanical strength at 4 weeks but superior strength at 8 weeks.

## Introduction

One of the most common postoperative complications in the mobile joint is adhesion that leads to stiffness [1]. Especially in rotator cuff repair, postoperative stiffness dissatisfied both patient and physician regardless of good healing of the repaired rotator cuff.

Improvement of surgical technique, early rehabilitation, and application of anti-adhesive material into the operation site are strategies to reduce adhesion after surgery. Minimizing invasiveness of surgery, for example arthroscopic surgery, is helpful to reduce surgical trauma. However, surgical technique alone is not sufficient to prevent adhesion and still postoperative stiffness is one of the most common complications in arthroscopic rotator cuff repair. Therefore, to reduce postoperative stiffness, numerous types of materials, including polylactic acid film, medical chitosan, medical sodium hyaluronate gel, polyethylene glycol berberine liquid, collagen, and gelatin form, have been developed to apply to the operation site to prevent adhesion [1, 2]. Among those materials, currently, solution type gel is the most widely used material in rotator cuff repair for its convenience [3, 4]. However, it can be easily moved and washed out in actively moving organ, such as the heart and joint, because of low viscosity [2]. Especially, in shoulder arthroscopic surgery, the agent could be hardly remaining in the expected site due to drain out of infused saline during the immediate postoperative period. Poloxamer-based thermosensitive hydrogels can be transformed from solution to gel in response to body temperature, due to their self-assembly in micelles [5, 6]. It stays as a solution at room temperature and changes as gel with higher viscosity in body temperature. Recently, its characteristic of rapid transition from sol to gel has been applied as drug delivery system and anti-adhesive material [7, 8].

However, there is a big dilemma that exists in the application of anti-adhesive agent which reduces fibrosis in orthopedic surgery, especially, in tendon repair, since the process of fibrosis partially contributes tendon healing, anti-adhesive agent may cause delay of tendon healing after tendon repair due to its effect of prevention of fibrosis. Up to date, there is no exact information about anti-adhesive agent usage in rotator cuff repair, although its usage is increasing.

The purpose of this study was to evaluate anti-adhesive effect and influence on the healing of rotator cuff repair when poloxamer-based thermosensitive gel is applied on acute rotator cuff repair in a rabbit model. We hypothesized that local administration of thermosensitive hydrogels in the repaired rotator cuff tendon prevent adhesion, but may affect tendon healing.

## Methods

The study was approved by the Institutional Animal Care and Use Committee at the Clinical Research Institute of the authors’ institute (IACUC no. 15-0010-C1A0). The experiments were performed on skeletally mature, New Zealand white male rabbits (24 weeks old, 3.5–4.0 kg).

### Allocation of experimental animals

This was a controlled animal study using a rabbit rotator cuff repair model in both shoulders. Each side of the shoulders was randomly selected, and the local application of the anti-adhesive agent (applied side) and saline to the contralateral side (control side) at the repair site was performed. Sample size analysis of the paired *t* test was based on ultimate failure load (mean difference of 50 N, standard deviation of 40 N, α-error 0.05, β-error 0.2, dropout rate 10%) [9]. Finally, a sample size of 8 was required for biomechanical studies to determine a significant difference in ultimate failure load. In addition, histologic analysis was done in another eight animals to evaluate tendon healing and adhesion in the bursal area. The influence of time on ultimate tensile strength of repaired tendon and histologic tendon healing and adhesion was evaluated at 4 and 8 weeks postoperatively. Therefore, 32 rabbits were included in the study.

### Poloxamer-based thermosensitive gel

Among various anti-adhesive agents, we have used a poloxamer 407/188-based thermosensitive gel (Mediclore®, CGbio Co., Ltd. Seongnam-si, Korea) [10]. The agent is a complex consisting of poloxamer 407/188, chitosan, and gelatin. Chitosan is a hemostatic polysaccharide gel obtained from crustaceans [11]. It has anti-adhesion efficacy by reducing scar formation by inhibition of proliferation of fibroblast [12]. It is known to have an anti-inflammatory effect by way of knockdown of inflammatory cytokines [13, 14]. Gelatin is also well known to have anti-adhesion potential [15].

### Surgical technique

All surgical procedures were performed by the same surgeon (K.S.H.). Anesthesia was induced by an intramuscular injection of 15 mg/kg zolazepam (Zoletil; Virbac S.A., Carros, France) and 5 mg/kg xylazine hydrochloride (Rompun; Bayer HealthCare, Leverkusen, Germany). Before procedures, 30 mg/kg prophylactic cefazolin (Cefamezin, Dong-A Pharm, Seoul, Korea) was administered. After thorough shaving and skin preparation, a sterile drape was applied to the area.

An anterior longitudinal incision was made at the shoulder, and the delto-pectoral interval was identified and split. The supraspinatus tendon, posterior to the bicipital groove and anterior to the scapular spine, was identified. Insertion of the supraspinatus at the greater tuberosity was sharply released using a scalpel. Decortication of the greater tuberosity was performed using a small burr.

Transosseous repair was done after two bone tunnels were created using a microdrill. Two transosseous simple stitches were made with two 3-0 nylon sutures (Ethicon, Johnson and Johnson, Somerville, NJ, USA). The same procedure was performed on the contralateral shoulder. After the repair was completed, either side of the shoulder was randomly selected and applied of 1 cm^3^ of Mediclore® (CGbio Co., Ltd. Seongnam-si, Korea) at the repair site (applied side). The same amount of the saline was infused to the contralateral repair (control side).

The fascia and subcutaneous tissue were sutured using interrupted 3-0 Vicryl suture (Ethicon, Johnson and Johnson, Somerville, NJ, USA), and the skin was sutured using interrupted 3-0 nylon suture. On the day of the operation and the first three postoperative days, the rabbits were given a subcutaneous injection of 0.2 mg/kg meloxicam (Metacam; Boehringer Ingelheim, Ingelheim am Rhein, Germany) for analgesia. An intramuscular injection of 30 mg/kg cefazolin was also administered once a day as a prophylactic antibiotic. The limbs were not immobilized postoperatively, and the rabbits were allowed to mobilize as desired in their respective cages.

### Specimen harvestings

At 4 and 8 weeks postoperatively, 16 rabbits at each time point were fully anesthetized and euthanized with carbon dioxide. In specimen for histologic analysis, the deltoid is remained with the underlying supraspinatus and humeral head. In specimen for biomechanical testing, the entire supraspinatus muscle and tendon was elevated from the suprascapular fossa along with the humeral head.

### Biomechanical evaluation

Mode of failure and the load to failure were evaluated at a rate of 1 mm/s with a preload of 5 N using a custom-fixture clamping system and an Instron 5565A materials testing machine (Instron, Norwood, MA, USA). The tendon was loaded until it separated from the bone or ruptured at its mid substance. For tensile testing, we designed and manufactured a custom clamping system. The system consists of two separate fixtures: a humeral head fixation unit, which rigidly fixes the humeral head and permits the supraspinatus tendon and muscle attached to the humeral head to come out through a hole, and a tendon fixation unit, which secures the myotendinous junction of the tendon. The supraspinatus tendon was fixed to this system along its anatomic direction to allow the tensile loading and tendon-bone interface to form a right angle. Data from the tensile load-to-failure testing were automatically collected using a computer-based data acquisition system [16].

### Histological analysis

Immediately after removing unnecessary soft tissue, specimens were fixed in neutral-buffered 10% formalin (pH 7.4) and decalcified. Paraffin blocks made in the repaired site 1-mm-wide serial sections parallel to the supraspinatus tendon were cut and stained with hematoxylin–eosin and Masson’s trichrome. To minimize bias on the part of the observer, all examinations were performed in a blinded fashion. The cross-section was examined under a microscope (Olympus DP 70, Tokyo, Japan) and analyzed using a system consisting of a video camera, automatic image analyzer, and image software (Image-Pro Plus, Media Cybernetics Inc., Bethesda, MD). Assessment was done by a pathologist who was not involved in the study. To evaluate the maturity of tendon-to-bone insertion quantitatively, we modified the tendon maturing scoring system reported by Watkins et al. (Table 1) [17, 18].

**Table 1:** The tendon maturing score [17]

	1	2	3	4
Cellularity	Marked	Moderate	Mild	Minimal
Fibrocytes	25%	25~50%	50~75%	75%
Vascularity	15 BV/low PF	11~15 BV/low PF	6~10 BV/low PF	6 BV/low PF
Cells parallel	25%	25~50%	50~75%	75%
Fibers parallel	25%	25~50%	50~75%	75%
Fiber diameter	25%	25~50%	50~75%	75%
Insertion histologic findings	C (+), R (−)	C (+), R (+), F (−)	C (+), R (+), F (+), Tidemark (−)	C (+), R (+), F (+), Tidemark (+)

*BV* blood vessel, *PF* power field, *C* continuity, *R* regularity, *F* fibrocartilage

**Fig. 1:**
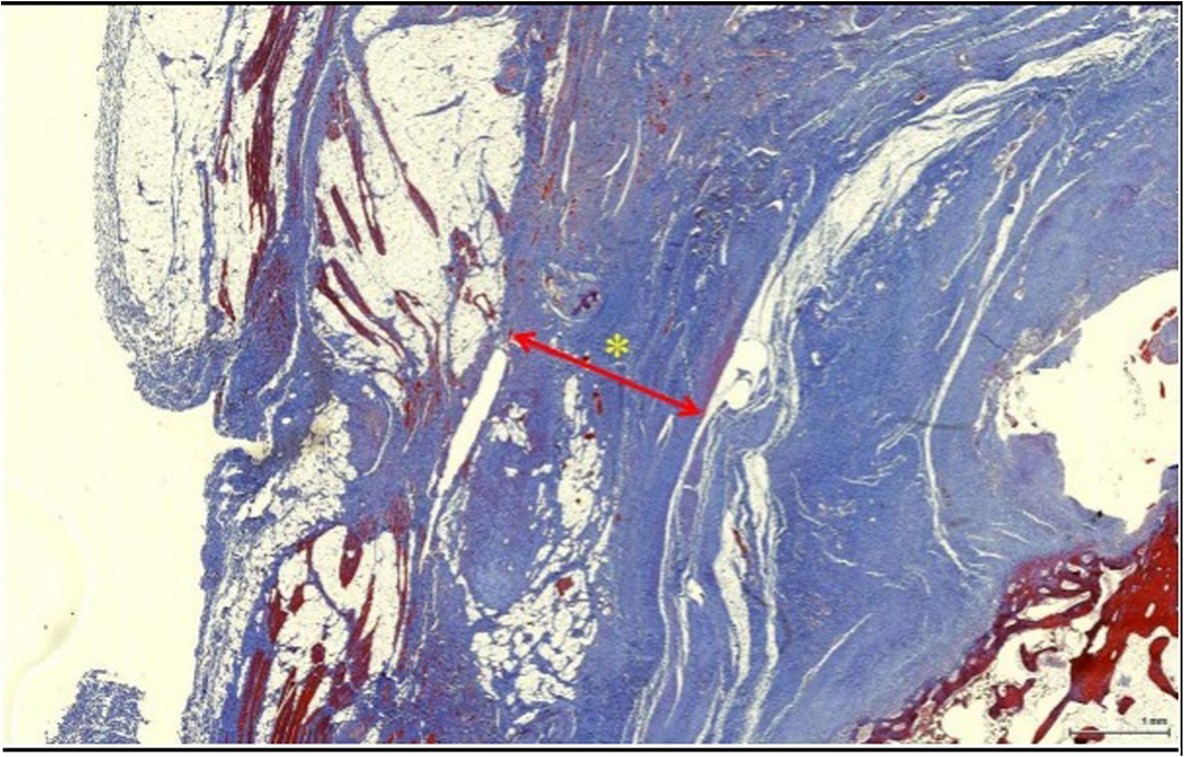
Thickness of fibrosis measurement in Masson’s trichrome stain. The thickness of the fibrosis (red line) between the supraspinatus and deltoid was measured with image software

To assess the degree of adhesion, we evaluated the thickness of fibrosis using Leica LAS EZ software (Leica Microsystems, Bannockburn, IL) between the deltoid muscle and rotator cuff tendon (Fig. 1).

### Statistical analysis

The paired *t* test was performed to determine the difference in biomechanical testing and depth of fibrosis between groups. Fisher’s exact test was used to evaluate the mode of failure in biomechanical testing. A Mann–Whitney test was used to examine the tendon score between groups. Statistical analyses were performed using IBM SPSS Statistics 21.0 software (IBM Corp, Armonk, NY, USA), and a *P* value of < 0.05 was taken as the level of statistical significance.

## Results

No experimental animals died before euthanasia. Therefore, at each time point, eight rabbits were included in biomechanical testing, and eight rabbits were included in histologic analysis.

### Biomechanical evaluation

The failure load at 4 weeks was lower in the applied side (Table 2); however, the failure load at 8 weeks was significantly higher in the experimental group than in the control side (*P* = 0.002). The mode of failure was not statistically different at 4 and 8 weeks (Table 3).

**Table 2:** Results of biomechanical testing

Time periods	Applied side (N)	Control side (N)	*P* value
4 weeks	95.2 ± 19.6	110.0 ± 20.5	0.017
8 weeks	148.3 ± 16.2	122.4 ± 16.9	0.002

**Table 3:** Mode of failure in biomechanical testing

Time periods	Applied side (M:I)	Control side (M:I)	*P* value
4 weeks	5: 3	3:5	0.619
8 weeks	4: 4	4:4	1.00

*M* mid-substance failure, *I* insertion failure

### Histologic evaluation

The tendon maturing score at 4 weeks and 8 weeks was not statistically different between the two groups (Table 4). The thickness of the fibrosis between the rotator cuff tendon and deltoid was thinner in the experimental group in both time points (Table 5). In other words, fibrosis at the subacromial space is much less in anti-adhesive agent applied side compared to the control side.

**Table 4:** The results of the modified tendon maturing score

Time periods	Groups	Score	*P* value
4 weeks	Applied side Control side	16.0 ± 0.7 15.7 ± 0.7	0.878
8 weeks	Applied side Control side	22.7 ± 0.4 20.7 ± 0.4	0.105

A perfect score in the scoring system is 28 points

**Table 5:** The results of the thickness of fibrosis between the rotator cuff tendon and deltoid

Time periods	Groups	Thickness (mm)	*P* value
4 weeks	Applied side Control side	0.50 ± 0.25 1.27 ± 0.47	0.002
8 weeks	Applied side Control side	0.37 ± 0.35 1.39 ± 0.50	0.003

## Discussion

The results of this study showed that the ultimate failure load was lower for the experimental group which is the application of a thermosensitive gel containing chitosan and gelatin than for the control group in the 4th week, whereas this load was higher for the experimental group than for the control group in the 8th week. This result may suggest that the healing process of rotator cuff tendon progressed slower with the application of the anti-adhesive agent. Excessive proliferation of fibroblasts may cause adhesion, but this proliferation is an essential process of tendon repair. Chitosan and gelatin, which are major ingredients of the anti-adhesion agent used in this study, are known to promote the proliferation of epithelial cells and endothelial cells but inhibit the proliferation of fibroblasts. Therefore, the tendon healing process may have been delayed due to inhibition of fibroblast proliferation during the first few weeks when the anti-adhesion agent is retained within the body. However, the increasing pattern of ultimate failure load over time showed that it increased in the experimental group and the control group by 155.77% and 111.27%, respectively, in the 8th week compared to the 4th week. Previous work has reported that the thermosensitive anti-adhesion agent is decomposed and excreted from the body by 4 weeks [19]. Therefore, the interpretation is possible that the experimental group showed a stronger physical strength than the control group after 4 weeks because the healing progressed without being affected by further inhibition due to the anti-adhesion agent. The high viscosity of hydrogel could be considered as another reason to explain this result. The highly viscous hydrogel may act as a scaffold around the tendon, thereby preventing fibrous ingrowth from the surrounding tissues [20]. On the other hand, chitosan also may play a major role in improving mechanical strength. Raftery et al. reported that addition of chitosan to a collagen scaffold enhanced chondrogenesis and osteogenesis, which could improve the mechanical properties of the collagen scaffold and consequently decrease the thickness of the fibrotic tissue [21]. Unlike the results of the mechanical strength experiment, the tendon maturing score showed a pattern that increased over time, with no differences between the groups at each point. These results show that the anti-adhesion agent does not inhibit tendon repair processes, including mechanical strength recovery and tissue maturation in the rotator cuff tear. Consequently, an anti-adhesive agent can successfully reduce the amount of fibrosis between the rotator cuff and the deltoid.

Postoperative adhesion is associated with fibroblast proliferation, and these fibroblasts transform into fibrotic scar tissues [22, 23]. One possible mechanism of fibrosis is that fibroblasts originating from surrounding tissues are brought by the blood circulation to the operation site, where they contribute to the progression of fibrosis [12]. Thus, fibroblast proliferation and hemostasis are the important targets for reducing postoperative adhesion. Restriction of fibroblast attachment to the operation site by application of a biophysical barrier is effective at preventing fibrosis [12]. Our results show that application of the thermosensitive gel containing chitosan successfully reduced the thickness of the fibrosis between the supraspinatus and deltoid. Because excessive fibrosis may result in adhesion, these results also mean that it fully plays a role as an anti-adhesive agent.

The thermosensitive anti-adhesion agent used in our study contains chitosan and gelatin. Chitosan has been reported to have properties that suppress fibroblast growth and collagenous fiber formation. Because it exhibits a hemostasis effect, it can also inhibit fibrin deposition. Moreover, chitosan is also known to inhibit transforming growth factor beta (TGF-b), which is involved in the development of fibrosis. For these various reasons, chitosan has been studied as a material for inclusion in anti-adhesion agents in the form of derivatives created in various forms through chemical modifications [24]. Gelatin, the other component of the thermosensitive anti-adhesion agent, shows strong adhesion to the wound surface. Like chitosan, gelatin also induces hemostasis and is known to promote the proliferation of epithelial cells and endothelial cells and inhibit the proliferation of fibroblasts [24].

The mechanism of the anti-adhesive effect of chitosan involves the regulation of Sirtuin1 (SIRT1), which inhibits apoptosis and the anti-inflammatory response of tenocytes [25]. The critical period for postoperative adhesion is within 5 to 10 days. Several authors have demonstrated that this time frame is independent of the size of the damaged surface [26]. The adhesion processes can be classified into several phases based on the chronological sequence of wound healing, which includes the exudative phase, resorptive phase, and reparative phase. In the exudative phase, a clot of coagulated blood covers the lesion on the surface, and this correlates with fibrin exudation. The resorptive phase corresponds to the time from 24 to 36 h, which is characterized by the migration of granulocytes, monocytes, and macrophages into the operation site and the formation of the fibrin clot [26, 27]. The subsequent reparative phase can last for weeks to months and consists of the formation of granulation tissue by the sprouting of capillaries and fibroblasts. In the case of peritoneal wound healing, this is an important stage, where either adhesion-free or adhesive wound healing will occur [26]. Thus, hydrogel-based barriers should cover the operation site efficiently to separate the site from neighboring tissue for as long duration as possible to prevent adhesion.

Our study has some limitations. First, acute rotator cuff repair in this rabbit model must be extrapolated to the situation of a human chronic rotator cuff tear. The different healing potential after surgery and different anatomic features between humans and rabbits limit the generalization of our results. Second, the operated limbs were not protected by immobilization. In clinical practice, the current trend is toward longer immobilization after rotator cuff repair. Both shoulders in our study were operated on to allow paired comparisons in each rabbit; therefore, immobilization was not possible. Immobilization of the operated limb could change the rate of healing [28]. However, because our study was a paired design, all conditions were the same for both the treated and control sides. Third, the study utilized an open rotator cuff repair model instead of an arthroscopic repair. As noted in the introduction, the majority of rotator cuff repairs in real situations is done using arthroscopic techniques, which use large quantities of infusion and irrigation fluids. Residual fluid and postoperative drainage of fluid may dilute and wash out the anti-adhesive agent applied at the operation site. Finally, this study only evaluated one type of anti-adhesive agent in rotator cuff repair. Therefore, the effect or influence on tendon healing could differ for other types of anti-adhesive agents. Thus, generalization of the results should be done with caution.

## Conclusion

Anti-adhesive agent in a rotator cuff model showed its effectiveness in reducing fibrosis in the subacromial space. In terms of influence in the healing of the tendon, interesting results were seen, in which the experimental group showed inferior biomechanical strength in 4 weeks, however, showed superior failure strength in 8 weeks.

## Data Availability

Not applicable.

## Abbreviations

SIRT1: Sirtuin1
TGF-b: Transforming growth factor beta
